# The Comparative Assessment of Effects on the Power System and Environment of Selected Electric Transport Means in Poland

**DOI:** 10.3390/ma14164556

**Published:** 2021-08-13

**Authors:** Katarzyna Markowska, Józef Flizikowski, Kazimierz Bieliński, Andrzej Tomporowski, Weronika Kruszelnicka, Robert Kasner, Patrycja Bałdowska-Witos, Łukasz Mazur

**Affiliations:** 1 Department of Logistics and Transport Technologies, Faculty of Transport and Aviation Engineering, Silesian University of Technology, 40-019 Katowice, Poland; Katarzyna.Markowska@polsl.pl; 2 Department of Machines and Technical Systems, Faculty of Mechanical Engineering, University of Sciences and Technology in Bydgoszcz, Al. Prof. S. Kaliskiego 7, 85-796 Bydgoszcz, Poland; fliz@utp.edu.pl (J.F.); a.tomporowski@utp.edu.pl (A.T.); werkur000@utp.edu.pl (W.K.); robert.kasner@gmail.com (R.K.); 3 Department of Telecommunications, Computer Science and Electrical Engineering, University of Sciences and Technology in Bydgoszcz, Al. Prof. S. Kaliskiego 7, 85-796 Bydgoszcz, Poland; kbiel@utp.edu.pl (K.B.); lukmaz003@utp.edu.pl (Ł.M.)

**Keywords:** electric vehicle, efficiency, transport performance indicators, life cycle assessment

## Abstract

Currently, electric vehicles are a rapidly growing alternative to those with combustion engines and can contribute to reduction of CO_2_ emissions in the transport sector, especially when the energy to power electric motors is predominantly derived from renewable sources. Until now, the comparison of environmental impact and influence of electric transport means on the power systems was not fully addressed in the case of Poland. The purpose of the study is to describe, analyse and assess electric vehicles (EV) operation against performance indicators in Poland, especially the influence of electric transport means (ETM) (electric cars, trams, trolley buses and buses) on power system and environment. The influence on the power system was investigated for the Polish National Powers system using the simulation of different scenarios of loads generated by EV charging. The energy demand of the National Power System and daily load variability indices were determined. Based on the data of ETM powers consumption and emissions of energy production, the emissions of harmful gases per one km and per one person were calculated, as well as the financial outlays for energy necessary to drive 1 km per 1 passenger. To assess and compare the environmental impact of the selected ETM life cycle, the life cycle assessment method was used. The results of environmental impacts were determined for selected assessment methods: CML 2 and IPCC 2013 GWP 100. The functional unit in this study is one selected ETM with a service life of 100,000 km. Comparison of trams, trolley buses, buses and electric passenger cars indicates that most beneficial are electric buses which do not need rails or overhead lines, thus investment costs are lower.

## 1. Introduction

The first model of an electric motor vehicle dates back to the early 19th century (specifically the year 1831, presented by M. Faraday) [1]. Decades later, at the end of the 1870s, the first electric railway designed by Werner von Siemens was presented at the Industrial Exhibition in Berlin. A few years later, he constructed a model of an electric tram and a trolley bus. Although the combustion engine vehicles developed more rapidly in the 20th century, it is the electric drive that occupies a dominant position in railway vehicles.

The number of electric vehicles in Europe is constantly growing. Actions are being taken to introduce this type of vehicles into public transport systems [2,3]. These actions include, e.g., involvement of legislation organs in favour of electric vehicles [4,5,6]. For example, in Poland, in 2017 the Electromobility Development Plan was introduced, assuming the development of infrastructure and industry for the purposes of electromobility. On the other hand, the Act of 11 January 2018 on electromobility and alternative fuels provides for a number of benefits for electric vehicle drivers, including: PHEV hybrid passenger cars, the possibility of using electric vehicles on bus lanes, additional parking spaces, an increase in the rates of depreciation charges and an exemption from certain fees, which is to encourage citizens to purchase electric cars [7]. In Germany, support programs have been introduced, including subsidies for companies and private sector, for the installation of charging stations and the purchase of electric cars. There are also exemptions and tax breaks [8]. Spain also provides grants for purchasing the electric vehicles and exempt from paying the car registration tax is available. Some other incentives could be found regional in Spain [9].

Infrastructure necessary for electric vehicles to operate has been developing for the last few years [10]. Unfortunately, high prices of electric cars are an obstacle to popularization of these vehicles. The biggest cause of such high prices are expensive technical solutions used for energy accumulation (costs of batteries). For this reason, many researchers have conducted research to improve the capacity of batteries used in electric cars, which is reflected by a large number of publications dealing with modifications of batteries and fast methods of charging [2,11,12]. Both, an increase of the energy density of batteries (an energy density of lithium batteries may reach as much as 200 Wh/kg) and a significant reduction of unit costs of batteries (currently, USD 190–250/kWh—it is planned to reduce them to USD 100/kWh) would make electric vehicles more attractive [13,14]. Electric vehicles are more eco-friendly, their motors are less power consuming and they are capable of energy recovery from braking [15].

New solutions should be devised to improve the already high energy efficiency of drives and the range of electric vehicles shortening the battery charging time. The capacity and charging time of batteries are still no match for the refuelling time in combustion vehicles.

Efficiency is one of operational criteria relating to the organisation, course and acquisition of useful and useless products of operation, in this case, electric transport. Efficiency is defined as a situation in which a person, company, factory, etc. uses resources such as time, materials or labour well, without wasting any. In relation to machines and devices it is understand as the difference between the amount of energy that is put into a machine in the form of fuel, effort, etc., and the amount that comes out of it [16].

Factors influencing the efficiency of electric transport are divided into technical and non-technical factors [17]. Technical factors include aspects relating to technical and operational parameters of vehicles, i.e., dimensions, weight, number of seats, technical data of the drive system, range, etc. Non-technical factors are classified into three categories [17]:

1. Economic factors—cost of operation, repair, insurance, etc.;
2. Organisational factors—organisation of a way, line and stops, optimal selection of a transport mode for a line, etc.;
3. Social factors—to include society structure, vehicle age, aesthetic impression (vehicle appearance), innovation (access to WiFi hotspots in vehicles), safety, etc.

Electric vehicles are considered more environmentally friendly than cars equipped with an internal combustion engine due to lack of direct emissions into the environment. They, however, require supply of electrical energy whose production is burdened with harmful emissivity [18]. Use of electric vehicles differs from use of vehicles with the combustion engine in terms of efficiency and performance. The research that has been carried out so far is connected with, e.g., assessment of environmental impacts of materials and elements in the life cycle of electric cars which is presented in [19]. Onat et al. [20] present a study of the environmental impact of electric cars powered by renewable energy sources and economic assessment based on LCC (Life Cycle Cost) method for the United States. Souza et al. [21] have made a comparative analysis of vehicles powered by fossil fuels, hybrid cars and electric cars for Brazil. Similar issues are addressed by the authors of work [22] who analyse the environmental impact of electric transport in Poland and the Czech Republic. Alves et al. [23] in turn, have developed a method for estimation of energy consumption by electric cars. There are relatively few analyses of electric transport in Poland. Those which are available address mainly carbon dioxide emissivity in the sector of transport in urban agglomerations [24,25,26].

Apart from economic and ecological effects, the growing number of electric vehicles, mostly the need to chargé them by using energy from power grid involves changes and disruptions in its functioning [27,28]. Many works deal with the issues of integrating charging electric cars with the power grid [28,29,30,31,32,33]. Bouallaga et al. [34] presents a methodology for electric vehicle charging management on the basis of genetic algorithms and Fuzzy–Boolean algorithm. Zhang et al. [35] propose a methodology for control of electrical grid frequency with charging stations. Ramos Munoz et al. [36] indicate the need to locate transformers in with vehicle charging stations. So far, the impact of charging stations in Poland on the power grid has not been analysed because of a small number of this type of facilities.

Basically, no publications have been found for comparison of the effects of different electric transport forms, e.g., electric cars, trams or trolley-buses, nor overall assessments of their environmental impact and influence of using and charging the electric vehicles on the power grid in Poland. Therefore, the authors of this study have undertaken an attempt to evaluate and compare a few forms of electric transport in terms of environmental impact and influence on the power system select Poland as regional border.

In the light of the above statements from the state of the art and technology the purpose of the article is to describe, analyse and assess electric vehicles operation against performance indicators in Poland. In this work the comparison of CO_2_ emission and other harmful gases and dust for electric cars, trams, trolley-buses and electric buses was presented, as well as the comparative life cycle assessment for selected transport means for 11 impact categories. The influence on the power grid was shown through a simulation of the electric car charging for four variants of 24 h load of power grid in order to indicate the most convenient time for charging.

The contribution of this paper is as follows:

- Indication of the impact of the appearance of a significant number of electric cars and the need to charge them on the Polish power systems;
- Estimation of emissions of harmful substances (CO_2_, SO_2_, NO_x_, CO and dust) related to the use of electric means of transport in Poland per road unit and per passenger, taking into account the Polish energy mix and various scenarios of the share of renewable energy sources in the electricity produced;
- Comparison of transport costs with the use of electric vehicles in Poland with an analysis;
- Performing a comparative analysis of the life cycle of selected means of transport with electric drive along with an indication of the critical impact areas, taking into account the Polish energy mix as a power source.

## 2. Condition of Electric Transport in Poland

Governmental forecasts concerning the development of the electromobility sector in Poland indicate that more than 1 million electric vehicles will be used by 2025 [37]. The idea of electromobility is associated mainly with electric vehicles. These are vehicles that use an electric drive and energy fed to the electric motor is supplied from the battery, e.g., a lithium-ion, nickel-metal hydride (Ni-MH) or nickel-cadmium battery (Ni-Cd).

The electromobility development plan provides a huge margin for development of both electric vehicles and public transport. In line with the governmental electromobility development plan in Poland, many Polish cities and communes have signed a letter of intent regarding the purchase of 780 electric buses by the end of 2020 [38]. The Act of 11 January 2018 on Electromobility and Alternative Fuels intensifies the development of electric buses in Poland—pursuant to Article 36 of the said Act, local self-government entities with the number of inhabitants not exceeding 50,000, should provide communication services or have them provided by an entity that uses at least 30% of zero emission buses within the relevant area (the Act defines a zero emission bus as a bus that uses hydrogen-derived electric energy using fuel cells or sources that do not lead to emissions of greenhouse gases, e.g., renewable energy; a trolley bus is also deemed a zero emission bus) [39].

Ursus Bus S.A. or Solaris Bus & Coach are leading manufacturers of electric buses. Ursus City Smile 18M is considered the fastest charging electric vehicle [38,40]: 3 min of charging at 700 V and 625 A enable 1 h of continuous operation of a vehicle. Solaris Urbino 12 from Solaris Bus & Coach (Bolechowo-Osiedle, Poland) received the main prize for the best city bus of 2017 in the ‘Bus of the Year’ competition [38].

Trams and trolley buses are fed from a municipal network of overhead lines with 600 V DC using current collectors, i.e., pantographs. The difference between these transport modes is that trams run on rails (track gauge in Poland is 1000 or 1435 mm [41]), whereas trolley buses employ the bus design in their wheel system. An important component of a modern trolley bus and tram is an inverter—an electronic device used to convert DC energy into AC [42]. Starting resistors responsible for diverting energy and reducing voltage when starting and braking a vehicle are also an important component of the said transport modes. However, such solutions are very inefficient and result in losing great amounts of energy. Therefore, braking energy recovery systems are increasingly installed with containers to store such energy.

Trams operate in 12 cities and two metropolitan areas in Poland—the Upper Silesian Industrial Region (13 cities) and the Łódź Metropolitan Area (6 locations)—whereas trolley buses only operate in three cities: Gdynia, Lublin and Tychy [39,41].

Polish companies are key players on the trolley bus and tram manufacturing market. PESA Bydgoszcz S.A. (Bydgoszcz, Poland) is a leading company manufacturing and modernising railway vehicles, including trams. PESA trams operate in Polish, Russian, Ukrainian, Romanian, Bulgarian and Hungarian cities [43]. Solaris Bus (Bolechowo-Osiedle, Poland) is one of the largest manufacturers of trolley buses in Europe. Flagship Solaris Trollino buses (Solaris Bus & Coach, Bolechowo-Osiedle, Poland) are operated in the majority of European countries.

## 3. Materials and Methods

### 3.1. Simulation of the Impact of Electric Vehicles on the Polish Power Grid

On the one hand, a solution to the problem of qualification of positive and negative effects of the system with electric vehicles, mainly electric cars and buses, refers to power system loading and relief, and, on the other hand, to the innovation of solutions. A million electric vehicles that will be operated from 2025, fitted with batteries of the capacity of approximately 30 kWh charged in a 10-h cycle, will load the system with additional 3 GW at the currently available power of approximately 43.4 GW (as of 31 December 2017 [44]). Electric vehicles may constitute 7% of the power system load. What seems to be a threat may be an opportunity for development and a substantial increase of the positive efficiency of the whole system.

On the basis of daily power system load variability profiles, a number of indicators of variable positive effects can be determined to assess the variability of power system loads used in a classical analysis, e.g., [45]:

- Average daily load degree–*m_dsr_*, which is defined as:
  (1) $$m_{dsr}=\frac{P_{dsr}}{P_{d\max}}\;$$
- Energy consumption variability index–*m_d_*_min_,
  (2) $$m_{d\min}=\frac{P_{d\min}}{P_{d\max}}\;$$
  where *P_dsr_*—average daily load, MW; *P_d_*_min_—minimum daily load, MW; and *P_d_*_max_—maximum daily load, MW.

In order to picture the impact of electric vehicles on the Polish power grid a simulation was carried out to select an optimal vehicle charging period during 24 h. It was assumed that the number of electric vehicles was 1 million and the battery capacity was 30 kWh. System data of the energy demand by the National Power System dated 06.06.2018 was used for the analysis [44]. The Polish energy mix in 2018 was dominated by hard coal-approximately 47.8% and lignite—29%, RES—12.7%, gaseous fuels—7.5% and others—3% [46]. Four concept variants were established to determine the most advantageous distribution of charging over time:

- First variant—vehicles will be charged from 12:00 at night to 10:00 a.m.;
- Second variant—charging will be spread out over rush hours from 07:00 a.m. to 05:00 p.m.;
- Third variant—the charging process moved to picture the night low period, i.e., from 10:00 p.m. to 8:00 a.m.;
- Forth variant—vehicles will be charged evenly round-the-clock.

### 3.2. The Assessment of the Electric Vehicles Environmental Impact

In assessment of variable negative effects of the electromobility system, the following indicators are taken into consideration:

- Unit CO_2_ emission yield per 1 km, *q_CO_*_2_:
  (3) $$q_{CO_{2}}=\frac{m_{CO_{2}}}{l_{T}}\;$$
- Unit SO_x_ emission yield per 1 km, *q_SOx_*:
  (4) $$q_{SO_{x}}=\frac{m_{SO_{x}}}{l_{T}}\;$$
- Unit NO_x_ emission yield per 1 km, *q_NOx_*:
  (5) $$q_{NO_{x}}=\frac{m_{NO_{x}}}{l_{T}}\;$$
- Unit CO emission yield per 1 km, *q_CO_*:
  (6) $$q_{CO}=\frac{m_{CO}}{l_{T}}\;$$
- Unit micro- and nano-dust yield per 1 km, *q_D_*:
  (7) $$q_{D}=\frac{m_{D}}{l_{T}}\;$$
  where *m_CO_*_2_—equivalent weight of CO_2_ emitted during transport in g or kg; *m_Sox_*—equivalent weight of SO_x_ emitted during transport in g or kg; *m_NOx_*—equivalent weight of NO_x_ emitted during transport in g or kg; *m_CO_*—equivalent weight of CO emitted during transport in g or kg; *m_D_*—equivalent weight of dust emitted during transport in g or kg; and *l_T_*—transport road in km.

Convenient indicators for assessment of negative effects of transport may be emission yields referring to a person, e.g., CO_2_ emission yield per transported person or kWh of consumed energy and its cost:

- Unit CO_2_ emission yield per person in a vehicle, *q_os_*:
  (8) $$q_{os}=\frac{m_{CO_{2}}}{l_{os}}\;$$
- Unit CO_2_ emission yield per 1 kWh of consumed electric power, *q_kWh_*:
  (9) $$q_{kWh}=\frac{m_{CO_{2}}}{l_{kWh}}\;$$
- Cost of consumed electric energy after driving 1 km per 1 passenger, *k_EE_*:
  (10) $$k_{EE}=\frac{K_{c}}{l_{km}}\;$$
  where *m_CO_*_2_—equivalent weight of CO_2_ emitted during transport in g or kg; *l_T_*—transport road in km; *l_os_*—number of transported persons, pcs; *l_kWh_*—amount of electric power consumed by vehicle, kWh; *k_EE_*—cost of electric energy consumed per 1 km of a transport road, PLN·km^−1^; and *K_C_*—total cost of electric energy consumed for transport of persons on a road *l*_km_, e.g., 100 km, in PLN.

#### 3.2.1. Emission during Operation of Electric Car for Different Electricity Mix

The analysis of emissions during the operation of an electric car was carried out on the example of a 2018 Nissan Leaf. The basic parameters of the car are presented in Table 1. The energy consumption of the analysed vehicle, in accordance with the cycle of WLTP (Worldwide Harmonized Light Vehicles Test Procedure) tests, is 20 kWh/100 km [1].

When assessing effects of operation of electric vehicles and cars, a few scenarios were considered: the supply by renewable energy sources with different share of cooperated RES installation (biogas and wind) (Table 2) and the supply by National Power System (KSE) with RES different shares (Table 3). The first seven rows in Table 2 present the CO_2_ emission per 1 kWh considering that energy comes only from renewable energy sources, with different share of electric energy form biogas plant and from wind power plant. The last three columns are the emissions of CO_2_ emission per 1 kWh considering energy mix (which include the mix of electric energy form fossil fuels and renewable energy sources) for Poland, China and Germany. An assumption was made that a renewable energy source, e.g., biogas, is fully controllable. To ensure self-sufficiency of a charging system based on renewable energy sources and independence from supply by an external power system, the power of a wind turbine and a biogas plant should be properly selected.

An assumption was made that a charging system (wind of 2 or 4 MW; biogas of 1 MW) with supply operates as a power co-operative. This is a type of a commune co-operative that is established to improve relations in the environment (reduce emissions), satisfy energy demands of members and electric vehicles and sell power/energy. Usually, energy co-operatives are associated with production of energy from renewable sources. Examples of such co-operatives are: ‘Bywind Energy Co-operative’ from the United Kingdom or ‘Nasza Energia’ from Poland. Emissivity of the Polish, Chinese and German power mix is given as references values in Table 2 and Table 3 [39,42]. The electricity mixes of different countries were included to show, that the results of transport emissivity will change regional, in dependence of electricity mix and share of renewable energy sources. This was meant to underline that using the same electric car in the different electricity production systems will cause the different emissions factors values per consumed kWh and travelled distance of one kilometre. The Chinese mix was chosen, because China is rapidly developing country and has the highest CO_2_ emissions in the world [48] and in 2020 was responsible for 53% of energy generated in the world from coal [49]. Germany has the greatest CO_2_ per year [48] and produce the most energy from coal from all European countries [49] (up to 2020).

The values of unit CO_2_ emission yield per person in a vehicle, *q_os_* (Equation (8)) and unit CO_2_ emission yield per 1 kWh of consumed electric power, *q_kWh_* (Equation (9)) were determined.

#### 3.2.2. Environmental and Cost Effects of Different Electric Transport Means

In order to assess and compare selected electric transport means, different types of vehicles were chosen and their basic technical data are given in Table 4.

The assessment includes:

- The comparison of the CO_2_, SO_2_, NO_x_, CO and dust emission related to production of electric energy that is needed for each transport mode to cover a distance of 1 km;
- The comparison of CO_2_ emission amount during production of electric energy necessary for travel by each transport mode per one passenger;
- The calculation of cost of travelling 100 km per 1 passenger for each type of vehicle;
- The comparative life cycle assessment of selected electric transport means.

##### The Emissions Related to Production of Electric Energy That Is Needed for Each Transport Mode to Cover a Distance of 1 km

The emission of CO_2_, SO_2_, NO_x_, CO and dust related to production of electric energy that is needed to supply the selected electric means of transport (Equations (3)–(7)) was calculated based on the data about vehicle energy consumption per kilometre (*Z_en,el_*, Table 4) and values of emissions of mentioned substances during energy production for Polish energy mix in 2016. Based on the report [58] the emission for produced energy unit was for CO_2_—781 kg/MWh, SO2—0.818 kg/MWh, NO_x_—0.824 kg/MWh, CO—0.252 kg/MWh and dust—0.053 kg/MWh.

##### Carbon Dioxide Emission Amount during Production of Electric Energy Necessary for Travel by Each Transport Mode Per One Passenger

The carbon dioxide emissions per one passenger per distance of 1 km travelled was calculated based on the emissions of CO_2_ obtained from calculation in point 3.2.3 and number of passengers travelling in the vehicle. For calculations the average number of passengers travelling by each transport means was used, it is: tram—80 passengers, trolley bus and electric bus—60, and electric car—2 (see Table 4).

##### Calculation of Cost of Travelling 100 km per 1 Passenger for Each Type of Vehicle

Operation costs, as significantly important in transport, depend on many factors which can be divided into [17]:

- Internal—type of a motor, travel style and dynamics, road conditions, car filling degree and car technical condition;
- External—fuel prices (for electric cars—prices of electric energy), prices of parts, toll fees, level of remuneration in transport companies, etc.

Based on electric energy consumption (Table 4), costs of travelling 100 km by each vehicle were estimated for one passenger. The data for costs in the Polish currency PLN were used for calculations, which were then converted into EUR, assuming the average EUR exchange rate of PLN 4.5877 (according to the data presented by the National Bank of Poland for the date 20 July 2021). It was assumed that the overhead line and electric bus charging stations are settled as per B21 tariff in which the price of electric energy is approximately EUR 0.09 (PLN 0.40/kWh). For electric cars, three supply options were considered in the analysis: using a single-phase socket at home–settlement as per G11 tariff (the energy price of EUR 0.12 (PLN 0.55/kWh) was taken), AC charging up to 22 kW from the charging station—the price of 1 kWh is EUR 0.26 (PLN 1.19) for this case [59], and quick DC charging—EUR 0.41 (PLN 1.89/kWh) [59].

##### The Comparative Life Cycle Assessment of Selected Electric Transport Means

The functional unit in this study is one passenger car with a service life of 100,000 km. The system boundary covers selected stages of the vehicle’s life cycle, combustion and operation [60]. The incineration stage includes the extraction, refining, transportation and distribution of fuels [61]. The geographical border of analysis was Poland. In order to determine the impact on the environment, the Life Cycle Assessment technique was adopted. SimaPro 8 software version 8.4. (PRé Sustainability, Amersfoort the Netherlands) was used to carry out the environmental analysis. The results of environmental impacts were determined for selected assessment methods: CML 2 and IPCC 2013 GWP 100. The Life Cycle Inventory (LCI) is a list of input/output data taken from the Ecoinvent databases.

## 4. Results and Discussion

### 4.1. Simulation of the Impact of Electric Vehicles on the Polish Power Grid

**Figure 1:**
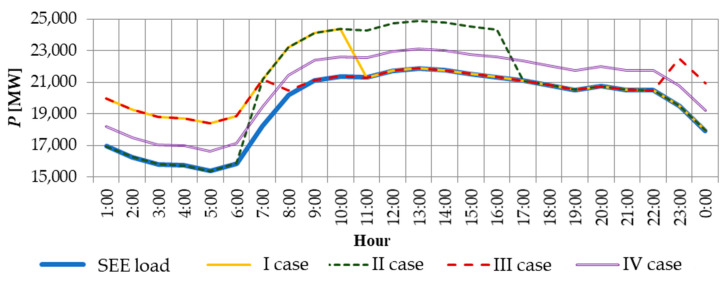
Comparison of the actual system load curve with simulated load variants of the system by charging stations.

Figure 1 shows results for simulated Polish power system load profile variants with 1 million used and supplied electric vehicles. Table 5 presents results of indices for four variants in question.

The average daily load degree, *m_ds_*_r_, and the energy consumption variability index, *m_dmin_*, indicate a power system load curve fitting degree—the closer the value to the unity, the more even the course of the curve [45]. From a power point of view, the most advantageous variant is a completely fitted load profile, since both energy losses and maximum load power losses are the lowest. However, such a solution is impossible to achieve. On the basis of results obtained, one can see that movement of the charging process to the night low period (3rd variant) brings the highest values of determined indices, thus the course of the curve will be the closest to the average load profile. In the second variant, it would be advisable to provide charging stations with individual power supply from, e.g., renewable energy sources to relieve the power system which has been suggested by Fathabadi et al. in [11]. It has been proven that supplying electric vehicle charging station with power from a hybrid system will allow for relieving the power grid [11].

The assumptions accepted in this study allow us to determine the period of electric car charging which is most advantageous for the power grid. Thus, all electric cars should be charged at night, which is rather difficult to do, due to the needs of electric cars users such as accessibility of charging stations and travel range of the cars. Similar conclusions are presented by Jain et al. in work [62], where they indicate that regulations of electric car charging should take into account such factors as: travelled distance, frequency of the vehicle charging and charging parameters.

Both development of electric vehicle charging infrastructure and storage of braking energy in containers, while considering the SmartGrid idea, fit in very well with the Vehicle to Grid (V2G) solution. The V2G interface, a telemetry and telecommunications engineering solution with a bidirectional energy flow: vehicles are charged in a so-called night low period of the power system load; in peak load periods, electric vehicles constitute a source of energy for the system. Vacheva et al. [63] indicate that electric cars have a large potential in V2G system to decrease load peak of the power grid. The research conducted by Iacobucci et al. [64] in Japanese price conditions shows that V2G solution is too expensive due to high prices of energy and batteries.

### 4.2. Emission during Operation of Electric Car for Different Electricity Mix

Table 6 presents the results of emissions of CO_2_ per one kWh consumed by electric vehicle and per one kilometre for scenario where the supply comes from the renewable energy sources with different shares of energy from biogas and wind power plant. As can be seen from Table 6, supplying the electric car with the energy produced form RES gives the smaller values of CO_2_ emissions connected with electric car operation. The emissions for different shares of energy from biogas and wind power plant suggests that the type of RES installation used for energy production have an influence on the CO_2_ emissions. When the energy for supplying electric car will in 100% come from the wind power plant the emissions factors per kWh and per kilometre will be the lowest from considered scenarios (4.7 gCO_2_/kWh, 0.9 gCO_2_/km, respectively), while for supply of 100% by biogas plant will be the highest (27 gCO_2_/kWh, 5.4 gCO_2_/km, respectively).

In case of co-supply by renewable energy sources and the National Power System, an equivalent of CO_2_ per road and energy emitted by vehicles powered by conventional fuels, i.e., diesel oil and petrol, was presented (Table 7).

On the basis of the analysis of data presented in Table 7, one can draw a conclusion that when an electric vehicle is supplied by the power mix in which less than 50% constitute energy form National Power System and rest from RES, the CO_2_ emissivity of electric vehicles is lower than for Diesel and petrol engine cars. Diesel-engine cars are characterised by a bit lower CO_2_ emissivity per road and energy unit than petrol-fuelled cars. With an increase of the share of renewable energy sources supplying electric drive vehicles, their CO_2_ emissions drop proportionally. For disabled supply from renewable energy sources, it is assumed that electric vehicles have a zero emissivity, whereas driving an electric vehicle in China causes a higher CO_2_ emissivity than in Poland and Germany. Similar statements were provided by Brockdorff at al. [65] on the basis of analysis of conditions in Malta. For comparison, Plotz et al. [66] indicate that the hybrid Chevrolet Volt, powered by energy from renewable sources, emits only 37 g CO_2_/km, which is lower than powering an electric vehicle from electrical grid with 70% share of RES energy (Table 7). In another work Plotz et al. [67] indicates that each extension of electric vehicle range by 1 km enables to decrease global emission of CO_2_ by 2–3%. Chen et al. [68], have made quite different observations regarding CO_2_ emissivity; they indicate that for the vehicles tested during movement or acceleration, CO_2_ emissions in their life cycle is higher for hybrid cars than for adequate vehicles powered by petrol.

Despite high emissivity connected with operation of electric vehicles Chinese mix, Liu et al. [69] show that long term effects of an increase in the number of electric vehicles involve a drop in CO_2_ emissivity and energy consumption in China as compared to the scenario without electric vehicles. Zhang et al. [70] indicate that the share and use of electric vehicles by the Chinese largely depends on the prices of electrical energy, capacity of batteries and the conditions of charging, as well as local incomes. Like Liu et al. [69], Teixeira et al. [71] have found out that replacement of combustion vehicles with electric ones in Brazil will cause reduction in carbon dioxide emission even with high emissivity from production of electrical energy to power them. Analysis performed by Trost et al. [72] shows that also in Germany introduction of electrical transport will contribute to reduction in CO_2_ emissivity in a long term perspective.

Hence, it needs to be noted that the results presented in Table 6 and Table 7, show consumption of fuels only at the stage of their operation, without considering the whole life cycle (production, operation and post life utilization). Analyses carried out for the whole life cycle of vehicles powered by electrical energy indicate that they are characterized by lower carbon dioxide emissivity throughout their life cycle than petrol powered vehicles [15,73,74,75,76].

### 4.3. Comparative Assessment of Environmental and Cost Effects of Different Electric Transport Means

#### 4.3.1. Emissions Related to Production of Electric Energy That Is Needed for Each Transport Mode to Cover a Distance of 1 km

Table 8 presents results of calculations of the amount of pollutants emitted to the environment due to generation of such amount of electric energy in power plants that is needed for each of the sample vehicles to cover a distance of 1 km.

Results given in Table 8 show that trams, the most energy consuming of all analysed transport modes (of the highest motor power), emit the greatest amounts of CO_2_, SO_2_, NO_x_, CO and dusts in an indirect way. The analysis of environmental and technical data does not provide a precise view of the situation and assessment of transport effects. Only when we look at social aspects, we can see a better image: trams, buses, trolley buses can carry much more passengers than passenger cars.

#### 4.3.2. CO_2_ Emission Amount during Production of Electric Energy Necessary for Travel by Each Transport Mode per One Passenger

**Figure 2:**
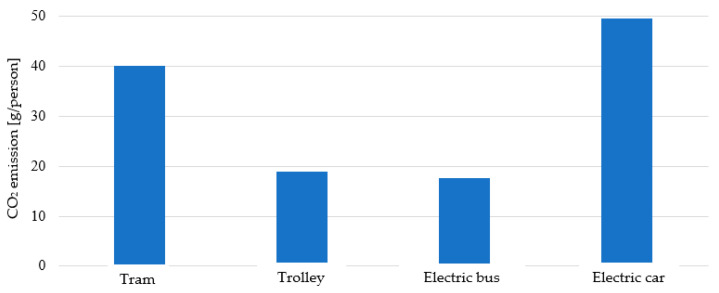
The amount of CO_2_ emission during production of electric energy necessary for travel by each transport mode per one passenger.

By referring the amount of pollutants per one passenger (Figure 2) to the number of passengers: tram—80, trolley bus and electric bus—60 and electric car—2, it turns out that an electric car is the most ‘emissive’ transport mode.

#### 4.3.3. The Cost of Travelling 100 km per One Passenger for Different Transport Modes

Table 9 shows the cost of travelling 100 km per 1 passenger for each vehicle (and the passenger car battery charging method).

As for the ecological assessment, electric buses and trolley buses proved to be the cheapest transport mode. The unit cost of electric car battery charging with so-called quick charging stations is more than three times higher than that of charging an electric car with a home system.

Comparing the cost of travel by diesel passenger car (for 100 km in the conditions of Polish roads per 1 passenger with an assumption of mean fuel consumption 5 L/100 km and price for one litre of ON EUR 1.11 (5.1 PLN) [77]) being EUR 1.11 for 100 km per one passenger, it can be said that it is higher than the same cost for an electric car powered from a single-phase socket at home, and lower than charged at the charging station or fast charging station. In Poland electric cars are too expensive, not only in terms of price but also operation and maintenance. Similar conclusion applies to China where electric vehicles are also burdened with too high costs to be used by an average user [70].

#### 4.3.4. The Comparative Life Cycle Assessment of Selected Electric Transport Means

Table 10 shows the cumulative emission levels of 11 impact categories: abiotic depletion, acidification, eutrophication, global warming (GWP100), ozone layer depletion (ODP), human toxicity, fresh water aquatic ecotoxicity, marine aquatic ecotoxicity, terrestrial ecotoxicity, photochemical oxidation of selected transport vehicles by road: transport by trolleybus, transport by tram, electric bus and electric car.

When starting the analysis under impact categories, particular attention was paid to assessing which of the ten considered categories may be the source of the greatest number of negative or positive environmental consequences in the life cycle and transport processes of selected road transport vehicles [78,79].

**Figure 3:**
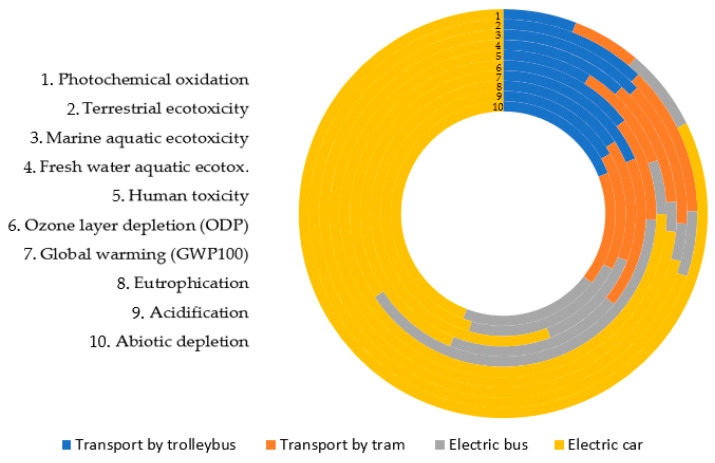
The results of characterizing the environmental impacts of electric cars.

It was noticed that the highest level of potential harmful effects on the environment, in the case of all tested objects, is characterized by one category: marine aquatic ecotoxicity 340.3688 kg 1.4-DB eq for electric car, 62.5702 kg 1.4-DB eq for transport by trolleybus, 61.78502 kg 1,4-DB eq for transport by tram and 15.91686 kg 1,4-DB eq for electric bus. The results of the remaining impact categories from the life cycle assessment point of view show a very low level of significance, hence no further assessment was carried out. Figure 3 presents the same data as the sum of all impact categories for various forms of transport: transport by trolleybus, transport by tram, electric bus and electric car. The greatest negative environmental impact was distinguished by transport by electric car. On the other hand, the smallest negative environmental damage emits from an electric bus.

Based on the emissivity of the electricity mix in Poland, electric car emissions are equal to 0.222771 kg CO_2 eq_/km (Table 11). Large rail vehicles had approximately 61% lower levels of potential CO_2 eq_/km emissions compared to passenger vehicles. Bus or trolleybus vehicles with extended range also have lower GHG than passenger cars, which allows to reduce CO_2 eq_/km emissions from up to 53 and 58%. The key factors affecting GHG emissions are energy consumption and GHG emissions per unit of electricity required.

## 5. Conclusions

Certainly, electric transport will develop rapidly over the next years. Determination of reasonable development directions requires objective indices of assessment of different transport solutions. The purpose of the article relating to objectivization of assessment of electric vehicles as per ecological, economic and power criteria has been achieved. Performance indicators of vehicles used in public transport, it is cars, buses, trolleys and trams were analysed as per: energy demand of the National Power System and daily load variability, vehicle use contexts, energy demand and consumption in different vehicles at 1 km of transport per person, amount of emitted harmful compounds during production of electric energy per 1 km, financial outlays for energy necessary to drive 1 km per 1 passenger. The influence of selected electric transport means on the environment was compared for 11 impact categories.

The cost analysis showed that the electric buses have the lowest cost of traveling 100 km per 1 passenger equal to EUR 0.19 compared to electric cars (ranging from EUR 0.76 to EUR 2.62 depending on the charging option), trams (EUR 0.44) and trolley bus (EUR 0.21). For the electric the emissions of toxic compound during production of one of electric energy that is needed for each transport mode to cover a distance of 1 km was the lowest (CO_2_ = 99.2 g/km, SO_2_ = 0.104 g/km, NO_x_ = 0.105 g/km, CO = 0.0320 g/km, dust = 0.00673 g/km), while the highest emissions were connected with trams (CO_2_ = 3202 g/km, SO_2_ = 3.354 g/km, NO_x_ = 3.378 g/km, CO = 1.033 g/km, dust = 0.217 g/km). However, when calculating the amount of CO_2_ emission during production of electric energy necessary for travel by each transport mode per one passenger the lowest CO_2_ emissions is connected with electric bus transport (17.573 g/per person) and the highest for electric cars (49.594 g/per person).

Based on the LCA results it was found that the highest level of potential harmful effects on the environment, in the case of all tested transport means, is characterized by one category: marine aquatic ecotoxicity 340.3688 kg 1.4-DB eq for electric car, 62.5702 kg 1.4-DB eq for transport by trolleybus, 61.78502 kg 1,4-DB eq for transport by tram and 15.91686 kg 1,4-DB eq for electric bus. The greatest total negative environmental impact was caused by transport by electric car and the smallest negative environmental damage by an electric bus. For the electric cars the greenhouse gas emissions during life cycle were also the highest (0.223 kg CO_2 eq_—IPCC method). On the other hand, the lowest greenhouse gas emissions are connected with tram transport (0.088 CO_2 eq_—IPCC method).

Assessment of tested electric transport modes for power, economic and ecological efficiency is ambiguous and depends on many variables. Therefore, it is difficult to compare public means of transport and electric cars. Electric cars provide better driving comfort and shorter travel times, although not always as they can get stuck in a traffic jam. This is where public transport, in particular trams, buses and trolley buses running in specially designated lanes come to the forefront. Individually, electric cars are vehicles which consume less electric energy; thus, unit costs of operation and emission of pollutants are lower. It is obvious, however, that electric means of public transport are of a greater carrying capacity which increases the efficiency of such vehicles.

Comparison of trams, trolley buses, buses and electric passenger cars indicates that the most beneficial are electric buses, which do not need rails or overhead lines, thus initial investment costs are lower. The following charging methods can be applied to electric buses: plug-in, inductive charging, and charging with a pantograph. This brings positive effects of charging buses both from a municipal network of overhead lines and using standard charging stations.

## Figures and Tables

**Table 1:** The technical data of Nissan Leaf 2018 [47].

Parameter	Value
Layout and boost	electric
Fuel Type	electricity
Maximum power	150 KM
Torque	320
Drive type	front-wheel drive
Front brakes	Disc brakes
Rear brakes	Disc brakes
Front suspension	McPherson columns
Rear suspension	Torsion beam
Wheels, front tires	185/50 R16
Wheels, rear tires	205/45 R16
Body type	hatchback
Number of doors	5
Own weight	1543 kg
Capacity	452 kg
Length	4490 mm
Width	1788 mm
Height	1530 mm
Wheelbase	2700 mm
Luggage capacity	435 L
Acceleration 0–100 km/h	7.9 s
Maximum speed	144 km/h
Range	378 km

**Table 2:** Type of a mix: wind/biogas and CO_2_ emissivity for electricity production [10,50].

No.	Biogas Plant Share (%)	Wind Power Plant Share (%)	Emission (gCO_2_/kWh)
1.	0	100	4.7
2.	10	90	6.9
3.	30	70	11.4
4.	50	50	15.9
5.	70	30	20.3
6.	90	10	24.8
7.	100	0	27.0
8.	Electric drive, Polish mix of the National Power System		650
9.	Electric drive, Chinese mix		712
10.	Electric drive, German mix		410

**Table 3:** Emissions for energy production for different shares of RES in polish National Power System [13].

No.	Renewable Energy Source Share (%)	National Power System Share (%)	Emissivity (gCO_2_/kWh)
1.	0	100	650
2.	10	90	585
3.	30	70	455
4.	50	50	325
5.	70	30	195
6.	90	10	65
7.	100	0	0
8.	Electric drive, Chinese mix		712
9.	Electric drive, German mix		410
10.	Diesel		291
11.	Petrol		316

**Table 4:** Examples of electric vehicles with their specification [51,52,53,54,55,56,57].

Specification	Unit	Tram	Trolley Bus	Electric Bus	Electric Car
		Pesa Swing 120 Na	Solaris Trollino 18	Solaris Urbino 12 Electric	Volkswagen e-Golf
Length	mm	19,350	18,000	12,000	4270
Width		2350	2550	2500	1799
Height		3400	3450	3250	1482
Motor	-	asynchronous	asynchronous	asynchronous	synchronous with permanent magnets
*P_max_*	kW	4 × 105	250	160	100
*v_max_*	km/h	70	65	50	150
Range	km	-	-	150	300
Max number of passengers	persons	122 (44 seats)	83 (40 seats)	99 (39 seats)	5
Average number of passengers	person	80	60	60	2
Supply	-	overhead line 0.6 kV DC	overhead line 0.6 kV DC	Li-Ion 210 kWh batteries	Li-Ion 35.8 kWh batteries
*Z_en.el_* ^1^	kWh/km	4.10	1.45 ^1^	1.35	0.127

^1^ Energy consumption refers to vehicles running in the network, without considering battery charging and energy recuperation [51].

**Table 5:** Exemplary daily load variability indices for various variants regarding the distribution of electric vehicle charging within 24 h.

Variant	*m_dsr_*	*m_dmin_*
Power system load	0.891	0.703
1st variant	0.851	0.736
2nd variant	0.834	0.618
3rd variant	0.917	0.817
4th variant	0.898	0.719

**Table 6:** Emissions of CO_2_ for Nissan Leaf per kWh consumed and distance of one kilometre travelled supplied by RES.

No.	Biogas Plant Share (%)	Wind Power Plant Share (%)	Emission (gCO_2_/kWh)	Emission (gCO_2_/km)
1.	0	100	4.7	0.9
2.	10	90	6.9	1.4
3.	30	70	11.4	2.3
4.	50	50	15.9	3.2
5.	70	30	20.3	4.1
6.	90	10	24.8	5.0
7.	100	0	27.0	5.4
8.	Electric drive, Polish mix of the National Power System		650	130
9.	Electric drive, Chinese mix		712	142
10.	Electric drive, German mix		410	82

**Table 7:** Type of a vehicle drive and effects of the CO_2_ emission in the environment per road and energy consumed.

No.	Renewable Energy Source Share (%)	National Power System Share (%)	Emissivity (gCO_2_/kWh)	Emissivity (gCO_2_/km)
1.	0	100	650	130
2.	10	90	585	117
3.	30	70	455	91
4.	50	50	325	65
5.	70	30	195	39
6.	90	10	65	13
7.	100	0	0	0
8.	Electric drive, Chinese mix		712	142
9.	Electric drive, German mix		410	82
10.	Diesel		291	118
11.	Petrol		316	122

**Table 8:** The amount of emitted compounds during production of electric energy that are needed for each transport mode to cover a distance of 1 km.

Emission Type	Unit	Tram	Trolley Bus	Electric Bus	Electric Car
CO_2_	g/km	3202	1132	1054	99.2
SO_2_		3.354	1.186	1.104	0.104
NO_x_		3.378	1.195	1.112	0.105
CO		1.033	0.365	0.340	0.0320
Dust		0.217	0.0769	0.07156	0.00673

**Table 9:** The cost of traveling 100 km per 1 passenger.

Supply Option	Electric Car		Tram		Trolley Bus		Electric Bus	
	EUR	PLN	EUR	PLN	EUR	PLN	EUR	PLN
single-phase socket at home	0.76	3.49	0.44	2.03	0.21	0.96	0.19	0.89
AC charging up to 22 kW from the charging station	1.65	7.56						
quick DC charging	2.62	12.00						

**Table 10:** The results of characterizing the environmental impacts of electric cars.

Impact Category	Unit	Total	Transport by Trolleybus	Transport by Tram	Electric Bus	Electric Car
Abiotic depletion	kg Sb eq	0.003753	0.000718	0.000617	0.00076	0.001658
Acidification	kg SO_2_ eq	0.003149	0.000532	0.000491	0.000695	0.001432
Eutrophication	kg PO_4_^−^ eq	0.001221	0.000194	0.000182	0.000164	0.000681
Global warming (GWP100)	kg CO_2_ eq	0.532437	0.099664	0.091444	0.107562	0.233767
Ozone layer depletion (ODP)	kg CFC-11 eq	5.13 × 10^−8^	7.48 × 10^−9^	5.65 × 10^−9^	2.06 × 10^−8^	1.75 × 10^−8^
Human toxicity	kg 1,4-DB eq	0.472225	0.042094	0.051008	0.025308	0.353815
Fresh water aquatic ecotoxicity	kg 1,4-DB eq	0.264432	0.031751	0.031476	0.007346	0.19386
Marine aquatic ecotoxicity	kg 1,4-DB eq	480.6409	62.5702	61.78502	15.91686	340.3688
Terrestrial ecotoxicity	kg 1,4-DB eq	0.00245	0.000301	0.000307	0.000133	0.00171
Photochemical oxidation	kg C_2_H_4_ eq	0.000384	2.23 × 10^−5^	2.08 × 10^−5^	2.5 × 10^−5^	0.000316

**Table 11:** The results of the analysis of the IPCC Global Warming Potential.

Impact Category	Unit	Total	Transport by Trolleybus	Transport by Tram	Electric Bus	Electric Car
IPCC GWP	kg CO_2 eq_	0.510858	0.094824	0.087545	0.105718	0.222771

## Data Availability

The data presented in this study are available on request from the corresponding author.

## References

[1] Wójtowicz S. (2012). Electric drive vehicles (in Polish: Pojazdy z napędem elektrycznym). Pr. Inst. Elektrotechniki.

[2] Xylia M., Silveira S. (2018). The role of charging technologies in upscaling the use of electric buses in public transport: Experiences from demonstration projects. Transp. Res. Part Policy Pract..

[3] Klauenberg J., Rudolph C., Zajicek J. (2016). Potential Users of Electric Mobility in Commercial Transport—Identification and Recommendations. Transp. Res. Procedia.

[4] Murawski J., Szczepański E. (2014). Prospects for electromobility development in Poland. Logistyka.

[5] Christensen L., Klauenberg J., Kveiborg O., Rudolph C. (2017). Suitability of commercial transport for a shift to electric mobility with Denmark and Germany as use cases. Res. Transp. Econ..

[6] Wang N., Tang L., Pan H. (2019). A global comparison and assessment of incentive policy on electric vehicle promotion. Sustain. Cities Soc..

[7] Gajewski J., Paprocki W., Pieriegud J. (2019). Elektromobilność w Polsce na tle Tendencji Europejskich i Globalnych.

[8] The Ultimate Guide to EV Incentives In Germany. https://blog.wallbox.com/en/the-ultimate-guide-to-ev-incentives-in-germany/.

[9] Everything You Need To Know About EV Incentives In Spain. https://blog.wallbox.com/en/spain-ev-incentives/.

[10] Benysek G., Jarnut M. (2012). Electric vehicle charging infrastructure in Poland. Renew. Sustain. Energy Rev..

[11] Fathabadi H. (2017). Novel grid-connected solar/wind powered electric vehicle charging station with vehicle-to-grid technology. Energy.

[12] Machura P., Li Q. (2019). A critical review on wireless charging for electric vehicles. Renew. Sustain. Energy Rev..

[13] Akumulatory i Nie Tylko. https://ep.com.pl/artykuly/10300-Akumulatory_i_nie_tylko.html.

[14] Carrilero I., González M., Anseán D., Viera J.C., Chacón J., Pereirinha P.G. (2018). Redesigning European Public Transport: Impact of New Battery Technologies in the Design of Electric Bus Fleets. Transp. Res. Procedia.

[15] Ahmadi P., Cai X.M., Khanna M. (2018). Multicriterion optimal electric drive vehicle selection based on lifecycle emission and lifecycle cost. Int. J. Energy Res..

[16] Efficiency. https://dictionary.cambridge.org/pl/dictionary/english/efficiency.

[17] Piątkowski P., Kraczkowski A., Surówka L. (2016). The analysis of public transport efficiency. Autobusy Tech. Eksploat. Syst. Transp..

[18] Cheng A.J., Tarroja B., Shaffer B., Samuelsen S. (2018). Comparing the emissions benefits of centralized vs. decentralized electric vehicle smart charging approaches: A case study of the year 2030 California electric grid. J. Power Sources.

[19] Sen B., Onat N.C., Kucukvar M., Omer T. (2019). Material footprint of electric vehicles: A multiregional life cycle assessment. J. Clean. Prod..

[20] Onat N.C., Kucukvar M., Afshar S. (2019). Eco-efficiency of electric vehicles in the United States: A life cycle assessment based principal component analysis. J. Clean. Prod..

[21] La Picirelli de Souza L., Silva Lora E.E., Escobar Palacio J.C., Rocha M.H., Grillo Reno M.L., Venturini O.J. (2018). Comparative environmental life cycle assessment of conventional vehicles with different fuel options, plug-in hybrid and electric vehicles for a sustainable transportation system in Brazil. J. Clean. Prod..

[22] Burchart-Korol D., Jursova S., Folęga P., Korol J., Pustejovska P., Blaut A. (2018). Environmental life cycle assessment of electric vehicles in Poland and the Czech Republic. J. Clean. Prod..

[23] Alves J., Baptista P.C., Goncalves G.A., Duarte G.O. (2016). Indirect methodologies to estimate energy use in vehicles: Application to battery electric vehicles. Energy Convers. Manag..

[24] Dzikuć M. (2017). Problems associated with the low emission limitation in Zielona Góra (Poland): Prospects and challenges. J. Clean. Prod..

[25] Dzikuć M., Adamczyk J., Piwowar A. (2017). Problems associated with the emissions limitations from road transport in the Lubuskie Province (Poland). Atmos. Environ..

[26] Bogacki M., Bździuch P. (2019). Urban bus emission trends in the Krakow metropolitan area (Poland) from 2010 to 2015. Transp. Res. Part Transp. Environ..

[27] Delgado J., Faria R., Moura P., de Almeida A.T. (2018). Impacts of plug-in electric vehicles in the portuguese electrical grid. Transp. Res. Part Transp. Environ..

[28] Aziz M., Huda M. (2019). Application opportunity of vehicles-to-grid in Indonesian electrical grid. Energy Procedia.

[29] Noel L., Papu Carrone A., Jensen A.F., Zarazua de Rubens G., Kester J., Sovacool B.K. (2019). Willingness to pay for electric vehicles and vehicle-to-grid applications: A Nordic choice experiment. Energy Econ..

[30] Morganti E., Browne M. (2018). Technical and operational obstacles to the adoption of electric vans in France and the UK: An operator perspective. Transp. Policy.

[31] Xiong Y., Wang B., Chu C., Gadh R. (2018). Vehicle grid integration for demand response with mixture user model and decentralized optimization. Appl. Energy.

[32] Druitt J., Früh W.-G. (2012). Simulation of demand management and grid balancing with electric vehicles. J. Power Sources.

[33] Tan K.M., Ramachandaramurthy V.K., Yong J.Y. (2016). Integration of electric vehicles in smart grid: A review on vehicle to grid technologies and optimization techniques. Renew. Sustain. Energy Rev..

[34] Bouallaga A., Davigny A., Courtecuisse V., Robyns B. (2017). Methodology for technical and economic assessment of electric vehicles integration in distribution grid. Math. Comput. Simul..

[35] Zhang Q., Li Y., Li C., Li C. (2018). Grid frequency regulation strategy considering individual driving demand of electric vehicle. Electr. Power Syst. Res..

[36] Ramos Muñoz E., Razeghi G., Zhang L., Jabbari F. (2016). Electric vehicle charging algorithms for coordination of the grid and distribution transformer levels. Energy.

[37] (2016). Ministerstwo Energii, Plan Rozwoju Elektromobilności w Polsce “Energia do Przyszłości”. Ministerstwo Energii. https://www.gov.pl/attachment/7cbc60f4-fec6-4dc1-b950-548cb0e52e9e.

[38] Skalski P. (2017). Electric buses in Poland. Autobusy Tech. Eksploat. Syst. Transp..

[39] (2018). Ustawa z Dnia 11 Stycznia 2018 r. o Elektromobilności i Paliwach Alternatywnych. https://isap.sejm.gov.pl/isap.nsf/download.xsp/WDU20180000317/T/D20180317L.pdf.

[40] Ursus City Smile 18M|Ursus. https://www.ursus.com/pl/produkt/ursus-city-smile-18m#basic-information.

[41] Beister M., Górny J. (2015). The development of the tramway infrastructure in Poland during accession to the European Union. TTS Tech. Transp. Szyn..

[42] Zalewska A. (2017). Structure and principle of operation of trolleybuse illustrated with an example of Solaris Trollino 12. Autobusy Tech. Eksploat. Syst. Transp..

[43] http://www.pesa.pl/.

[44] Zapotrzebowanie KSE-PSE. https://www.pse.pl/obszary-dzialalnosci/krajowy-system-elektroenergetyczny/zapotrzebowanie-kse.

[45] Chojnacki A.Ł. (2018). Analysis of daily, weekly and annual load variability of electricity in power networks of communal and industrial customers. Przegląd Elektrotechniczny.

[46] Struktura Produkcji Energii Elektrycznej w Czerwcu 2021 r. https://www.rynekelektryczny.pl/produkcja-energii-elektrycznej-w-polsce/.

[47] Nissan Leaf Udowadnia, że Elektrykiem Można Jeździć na co Dzień. Pod Jednym Warunkiem. https://autokult.pl/31582,nissan-leaf-udowadnia-ze-elektrykiem-mozna-jezdzic-na-co-dzien-pod-jednym-warunkiem,all.

[48] Global Carbon Atlas. http://www.globalcarbonatlas.org/en/CO2-emissions.

[49] Jones D. (2021). Global Electricity Review 2021. Global Trends. EMBER Coal to Clean Energy Policy. https://ember-climate.org/global-electricity-review-2021/global-trends/.

[50] Flizikowski J.B. (2011). Intelligent grinding system. Inżynieria i Aparatura Chemiczna.

[51] Bartłomiejczyk M. (2016). Practical application of In Motion Charging: Using trolleybuses on bus routes in Gdynia. Autobusy Tech. Eksploat. Syst. Transp..

[52] PSPA Katalog Pojazdów Elektrycznych BEV PHEV 2018. PSPA 2018. https://pspa.com.pl/media/2018/06/Katalog_pojazdow_elektrycznych_2018.pdf.

[53] Kuminek T. (2013). Energy consumption in tram transport. Logist. Transp..

[54] Połom M., Bartłomiejczyk M. (2011). Alternative drive sources in trolleybuses—Review of implemented solutions in the European cities. Transport Miejski i Regionalny..

[55] Solaris Bus & Coach S.A SOLARIS (2017). Napędy Alternatywne. Katalog Produktowy 2017/2018. https://www.solarisbus.com/pl.

[56] Solaris Urbino 12 Electric—SamochodyElektryczne.org. http://samochodyelektryczne.org/solaris_urbino_12_electric.htm.

[57] Grupa Urbino Electric—Solaris Bus & Coach S.A. https://www.solarisbus.com/pl/pojazdy/napedy-alternatywne-elektryczne-hybrydowe-hybryda/grupa-urbino-electric.

[58] (2017). IOŚ-PIB, Krajowy Ośrodek Bilansowania i Zarządzania Emisjami Wskaźniki emisyjności CO2, SO2, NOx, CO i Pyłu Całkowitego dla Energii Elektrycznej na Podstawie Informacji Zawartych w Krajowej Bazie o Emisjach Gazów cieplarnianych i Innych Substancji za 2016 rok. https://www.kobize.pl/uploads/materialy/materialy_do_pobrania/wskazniki_emisyjnosci/180108_wskazniki_spalanie_na_mwh.pdf.

[59] Adamczuk M. (2018). Stacje Ładowania aut Greenway Będą Płatne. Obliczamy Koszt Eksploatacji „Elektryka” Według Nowych Stawek. https://spidersweb.pl/autoblog/koniec-darmowego-ladowania-elektrykow/.

[60] Piasecka I., Baldowska-Witos P., Flizikowski J., Piotrowska K., Tomporowski A. (2020). Control the System and Environment of Post-Production *Wind Turbine* Blade Waste Using Life Cycle Models. Part 1. Environmental Transformation Models. Polymers.

[61] Baldowska-Witos P., Kruszelnicka W., Kasner R., Tomporowski A., Flizikowski J., Klos Z., Piotrowska K., Markowska K. (2020). Application of LCA Method for Assessment of Environmental Impacts of a Polylactide (PLA) Bottle Shaping. Polymers.

[62] Jain P., Das A., Jain T. (2019). Aggregated electric vehicle resource modelling for regulation services commitment in power grid. Sustain. Cities Soc..

[63] Vacheva G., Hinov N., Kanchev H., Stanev R., Cornea O. (2019). Energy Flows Management of Multiple Electric Vehicles in Smart Grid. Elektron. Ir Elektrotechnika.

[64] Iacobucci R., McLellan B., Tezuka T. (2019). Optimization of shared autonomous electric vehicles operations with charge scheduling and vehicle-to-grid. Transp. Res. Part C Emerg. Technol..

[65] von Brockdorff P., Tanti G. (2017). Carbon emissions of plug-in electric vehicles in Malta: A policy review. Case Stud. Transp. Policy.

[66] Plötz P., Funke S.Á., Jochem P. (2018). The impact of daily and annual driving on fuel economy and CO_2_ emissions of plug-in hybrid electric vehicles. Transp. Res. Part Policy Pract..

[67] Plötz P., Funke S.Á., Jochem P. (2018). Empirical Fuel Consumption and CO_2_ Emissions of Plug-In Hybrid Electric Vehicles. J. Ind. Ecol..

[68] Chen Y., Hu K., Zhao J., Li G., Johnson J., Zietsman J. (2018). In-use energy and CO_2_ emissions impact of a plug-in hybrid and battery electric vehicle based on real-world driving. Int. J. Environ. Sci. Technol..

[69] Liu F., Zhao F., Liu Z., Hao H. (2018). China’s Electric Vehicle Deployment: Energy and Greenhouse Gas Emission Impacts. Energies.

[70] Zhang Q., Ou X., Zhang X. (2018). Future penetration and impacts of electric vehicles on transport energy consumption and CO_2_ emissions in different Chinese tiered cities. Sci. China Technol. Sci..

[71] Teixeira A.C.R., Sodré J.R. (2018). Impacts of replacement of engine powered vehicles by electric vehicles on energy consumption and CO2 emissions. Transp. Res. Part Transp. Environ..

[72] Trost T., Sterner M., Bruckner T. (2017). Impact of electric vehicles and synthetic gaseous fuels on final energy consumption and carbon dioxide emissions in Germany based on long-term vehicle fleet modelling. Energy.

[73] Yu A., Wei Y., Chen W., Peng N., Peng L. (2018). Life cycle environmental impacts and carbon emissions: A case study of electric and gasoline vehicles in China. Transp. Res. Part Transp. Environ..

[74] Onn C.C., Mohd N.S., Yuen C.W., Loo S.C., Koting S., Abd Rashid A.F., Karim M.R., Yusoff S. (2018). Greenhouse gas emissions associated with electric vehicle charging: The impact of electricity generation mix in a developing country. Transp. Res. Part Transp. Environ..

[75] Wu Z., Wang M., Zheng J., Sun X., Zhao M., Wang X. (2018). Life cycle greenhouse gas emission reduction potential of battery electric vehicle. J. Clean. Prod..

[76] Requia W.J., Adams M.D., Arain A., Koutrakis P., Ferguson M. (2017). Carbon dioxide emissions of plug-in hybrid electric vehicles: A life-cycle analysis in eight Canadian cities. Renew. Sustain. Energy Rev..

[77] ON—Średnia Cena Oleju Napędowego w Polsce. https://nafta.wnp.pl/ceny_paliw/det_dis.html.

[78] Piasecka I., Baldowska-Witos P., Piotrowska K., Tomporowski A. (2020). Eco-Energetical Life Cycle Assessment of Materials and Components of Photovoltaic Power Plant. Energies.

[79] Baldowska-Witos P., Piotrowska K., Kruszelnicka W., Blaszczak M., Tomporowski A., Opielak M., Kasner R., Flizikowski J. (2020). Managing the Uncertainty and Accuracy of Life Cycle Assessment Results for the Process of Beverage Bottle Moulding. Polymers.

